# Examination of CD302 as a potential therapeutic target for acute myeloid leukemia

**DOI:** 10.1371/journal.pone.0216368

**Published:** 2019-05-10

**Authors:** Tsun-Ho Lo, Edward Abadir, Robin E. Gasiorowski, Karieshma Kabani, Murari Ramesh, Daniel Orellana, Phillip D. Fromm, Fiona Kupresanin, Elizabeth Newman, Ilona Cunningham, Derek N. J. Hart, Pablo A. Silveira, Georgina J. Clark

**Affiliations:** 1 Dendritic Cell Research, ANZAC Research Institute, Sydney, NSW, Australia; 2 Sydney Medical School, University of Sydney, Sydney, NSW, Australia; 3 Department of Haematology, Concord Repatriation General Hospital, Sydney, NSW, Australia; 4 Institute of Haematology, Royal Prince Alfred Hospital, Sydney, NSW, Australia; Emory University, UNITED STATES

## Abstract

Acute myeloid leukemia (AML) is the most common form of adult acute leukemia with ~20,000 new cases yearly. The disease develops in people of all ages, but is more prominent in the elderly, who due to limited treatment options, have poor overall survival rates. Monoclonal antibodies (mAb) targeting specific cell surface molecules have proven to be safe and effective in different haematological malignancies. However, AML target molecules are currently limited so discovery of new targets would be highly beneficial to patients. We examined the C-type lectin receptor CD302 as a potential therapeutic target for AML due to its selective expression in myeloid immune populations. In a cohort of 33 AML patients with varied morphological and karyotypic classifications, 88% were found to express CD302 on the surface of blasts and 80% on the surface of CD34^+^ CD38^-^ population enriched with leukemic stem cells. A mAb targeting human CD302 was effective in mediating antibody dependent cell cytotoxicity and was internalised, making it amenable to toxin conjugation. Targeting CD302 with antibody limited *in vivo* engraftment of the leukemic cell line HL-60 in NOD/SCID mice. While CD302 was expressed in a hepatic cell line, HepG2, this molecule was not detected on the surface of HepG2, nor could HepG2 be killed using a CD302 antibody-drug conjugate. Expression was however found on the surface of haematopoietic stem cells suggesting that targeting CD302 would be most effective prior to haematopoietic transplantation. These studies provide the foundation for examining CD302 as a potential therapeutic target for AML.

## Introduction

Monoclonal antibodies (mAb) and their derivatives such as antibody drug conjugates (ADC), bispecific T Cell engagers and chimeric antigen receptor T cells, are rapidly being developed as the next generation of anti-cancer treatments [1]. These therapeutic agents offer the advantage of high specificity and potency with the potential of limited toxicity due to their ability to recognise molecular targets on tumours [2]. Whilst advances have been made in the development of mAb based therapy in other haematological diseases such as B cell lymphoma [3] and multiple myeloma [4], progress in acute myeloid leukemia (AML) has remained unsatisfactory. An ideal AML target should be highly expressed on the surface of leukemic blasts with limited expression on healthy cells [5]. AML arises from haemopoietic stem cell (HSC) and multipotent progenitor populations (MPP) resulting in substantial overlap in surface molecule expression [6]. Additional properties including internalisation, induction of antibody dependent cell mediated cytotoxicity (ADCC) or functional repression are favourable for designing mAb therapeutic strategies. Despite ongoing work, no ideal AML target has been identified [5, 6]. Approximately 70% of patients under the age of 60 achieve complete remission following conventional treatment, but many relapse causing a 40% overall survival rate [7]. This is believed to be due to the persistence of leukemic stem cells (LSC), which are not eliminated efficiently with current treatments and re-populate over time [8–10]. Therefore, it is critical that an AML target molecule be expressed by LSC and blasts. Given the heterogeneous nature of AML, different targets could be required for the treatment of malignant cells represented by this disease.

We propose CD302 as a potential therapeutic target for AML. CD302 is the simplest type I transmembrane C-type lectin receptor (CLR) described [11]. The protein consists of 232 amino acids containing a single C-type lectin like domain. Amongst human leukocytes, CD302 is restricted to myeloid derived populations including monocytes, macrophages, dendritic cells and granulocytes. This expression profile led us to explore CD302 as a potential target for myeloid malignancies. CD302 colocalizes with f-actin rich filopodia, lamellopodia and podosomes in macrophage and transfected cell lines, indicative of a role in attachment or migration [11], a function subsequently confirmed by CD302 knockout mouse studies [12].

In the current study, we examined CD302 expression on leukemic cell lines and primary AML in comparison to HSC and monocytes from healthy donors. We explored the ability of anti-CD302 mAb to mediate ADCC and affect leukemic cell migration using *in vitro* and *in vivo* models. We have further characterised differences in CD302 distribution between AML and hepatocyte cell lines and demonstrated a proof of principal *in vitro* ADC model.

## Material and methods

### Preparation of tissue samples

Patient blood or bone marrow (BM) samples from patients with AML were collected at the Concord Repatriation General Hospital (CRGH) or Royal Prince Alfred Hospital (Sydney, Australia). Patients ranged from 16–95 years of age, and had blast percentages in sample that ranged from 10–95% at the time of collection (S1 Table). Healthy donor blood samples were collected, with informed consent, from a donor panel maintained by the Department of Haematology, CRGH. Blood was obtained by venesection and PMBC isolated by density gradient centrifugation using Ficoll-Paque Plus (GE Healthcare) with the manufacturer’s protocols. Cord blood (CB) samples were obtained from the Sydney CB Bank with mononuclear cells collected as above. BM aspirates were collected from the posterior iliac crest of patients and healthy volunteers. Samples were then passed through a 22G needle to disrupt BM fragments before proceeding to isolation of mononuclear cells as above. To purify human monocytes, healthy donor peripheral blood mononuclear cells (PBMC) were labelled with CD14 Microbeads (Miltenyi Biotec) and positively selected using an AutoMACS Pro (Miltenyi Biotec). Ethical approval for human studies was obtained from the Sydney Local District Human Research Ethics Committee (HREC/12/CRGH/59, HREC/11/CRGH/61 & 118).

### Cell lines

HL-60 and HEL cell lines were previously obtained by Professor Derek Hart at the Haematology & Immunology Research Group, Christchurch School of Medicine, University of Otago, New Zealand. The U937 and HS-5 cell lines was sourced from the American Type Culture Collection (ATCC). These lines were maintained in complete RPMI 1640 (supplemented with 10% FCS, 2mM Gluta-MAX, 100U/ml penicillin and 100μg/ml streptomycin; ThermoFisher). The HepG2 cell line was purchased from ATCC and grown in DMEM with 1g/l D-glucose, 10% FCS, 2mM Gluta-MAX, 100U/ml penicillin and 100μg/ml streptomycin (ThermoFisher).

### Gene expression

AML gene expression data was retrieved from the Gene Expression Omnibus microarray dataset GSE14468 [13]. The series matrix files were parsed in R and the probe ID and signal value corresponding to *CD302* (*203799_AT*) and *CD33* (*206120_AT*) extracted. Only samples containing French American British (FAB) AML subtype classification were analysed. For quantitative PCR (qPCR), total RNA from tissues or cells was extracted using TRIzol reagent and synthesized into cDNA using the SuperScript III kit (ThermoFisher) as per manufacturer’s instructions. Method and primers for human CD302 and HPRT qPCR are described in [12].

### Flow cytometry

AML and healthy PBMC samples were phenotyped with CD45-V500 (HI30), CD34-PE-CY7 (581), CD38-V450 (HB7) and CD33-PE (WM53), CD90-AF700 (5E10), CD45RA-APC-H7 (SH9), CD11c-APC-AF700 (B-Ly6) and HLA-DR-APC-H7 (L243) mAbs from BD Biosciences or BioLegend. The lineage (Lin) cocktail consisted of CD235a (GA-R2), CD14 (MφP9), CD20 (2H7), CD19 (HIB19), CD56 (NCAM16.2) and CD3 (SK7; BD Biosciences). The mouse anti-human CD302 IgG1mAb antibody (MMRI-20) was used unlabelled or as a PE conjugate [11]. The mAb CMRF-81, specific for tetanus toxoid, was used as the mouse IgG1 isotype control [14]. DAPI (3μM; ThermoFisher) staining was used to exclude dead cells. Data were collected on Accuri C6, Canto, Fortessa LSR or Influx flow cytometers (BD Biosciences) and analysed with FlowJo 10 software (Treestar). The gating strategy for identifying BM/CB HSC and MPP are shown in panel A of S1 Fig. Binding was displayed as a geometric mean fluorescence intensity (geoMFI) ratio which was calculated by the formula: geoMFI test antibody/geoMFI isotype control. A ratio of ≥3 was considered positive. T-distributed stochastic neighbour embedding (t-SNE) visualisation was performed on FlowJo 10.

### Western blot

Cell suspensions (2.5x10^7^ cells/ml) were solubilised in modified RIPA buffer (1% Triton X-100, 0.25% sodium deoxycholate, 0.15M NaCl, 50mM Tris-HCl, 5mM EDTA containing Protease Inhibitor (Roche, Basel, Switzerland)). Protein content was determined by bicinchoninic acid assay (ThermoFisher). Lysates (5μg) were fractionated on a 4–12% Bis-Tris gel (Bolt, ThermoFisher) under reducing conditions. Proteins were transferred to nitrocellulose (Novex Miniblot; ThermoFisher) using an iBlot. Membranes were stained with Ponceau before overnight incubation in 5%BSA in TBST. Membranes were incubated with 1μg/ml rabbit anti-human CD302 polyclonal antibody (LS-C119435; LS Bio) followed by 1:1000 HRP conjugated goat anti-rabbit IgG Fc antibody (A6154 Sigma-Aldrich). Protein bands were detected by chemiluminescence using Clarity Western ECL substrate (BioRad) and visualised on a BioRad GelDoc. Molecular weights (MW) of proteins were determined by comparison with Precision Plus standards (BioRad).

### Immunohistology

HepG2 or HL-60 cells (4x10^4^ cells/well) were adhered to Lab-Tek II Chamber Slide (ThermoFisher) for 30 min or overnight in 5% CO_2_ at 37°C, respectively. Cells were fixed with 4% paraformaldehyde and rehydrated using 1% BSA/PBS. Cells were blocked with 10% goat serum (Invitrogen) and stained with either MMRI-20 or isotype control (10μg/ml) for 30 min at 37°C. Goat anti-mouse (GAM) IgG-AF488 antibody (ThermoFisher) was used to detect primary antibodies. Phalloidin-AF594 and 18μM DAPI (ThermoFisher) were used to identify f-actin at the cell surface and the nucleus, respectively. Slides were imaged using a 3i VIVO Spinning Disc Microscope (Intelligent Imaging Innovations, Inc.) and analysed with Image J (NIH) software.

### Antibody internalisation assay

HL-60 cells were incubated with MMRI-20-PE or isotype control-PE (10μg/ml) on ice for 20 min. Antibody coated cells were then incubated at 37°C/5% CO_2_ for the indicated times to allow internalisation. After incubation, a secondary GAM IgG-AF488 antibody was applied to the samples for 20 min on ice to detect remaining surface antibody and compared to the total (surface and internalised) PE staining. Cells were fixed in 1% paraformaldehyde/PBS followed by flow cytometry analysis. Relative MFI was calculated as a percentage of staining at 0 min.

### Colony forming units (CFU)

Frozen CB cells were incubated with MMRI-20 mAb followed by GAM IgG-AF488 (Invitrogen). Subsequent staining with a Lin stain was performed. DAPI^-^ Lin^-^ CD302^+^ or CD302^-^ fractions were FACS isolated and resuspended in IMDM media (Stemcell Technologies). Equal numbers of each sorted fraction were plated at 1.5–2.5x10^4^ cells/plate in semi-solid methylcellulose medium (MethoCult Classic, Stemcell Technologies). Plates were cultured at 37°C and 5% CO_2_ for 12–14 days prior to counting of multi-lineage, myeloid and erythroid colonies in wells with a light microscope.

### ADCC

HL-60 or U937 target (T) cells labelled with 2.5μM Calcein-AM (ThermoFisher) as per manufacturer’s protocol and resuspended in complete RPMI 1640. Target cells (5x10^3^) were mixed with 5x10^4^ C57BL/6J female mouse (Animal Resources Centre, Perth, Australia) spleen effectors (E), 1000U of human IL-2 (Invitrogen) and the indicated concentrations of MMRI-20 or isotype control in triplicate. Plates were incubated for 18h at 37°C and 5% CO_2_. Cells were subsequently labelled with 3μM DAPI for 20 min to detect death of Calcein-AM^+^ target cells via flow cytometry. Spontaneous and maximal death was determined by culturing target cells alone or with 2% Triton-X, respectively. Cytotoxicity was calculated with the formula: E+T(antibody)–E+T(no antibody)/ T(max)–T(spontaneous).

### Migration assays

HL-60 and U937 were incubated with MMRI-20 or isotype control mAb (10 μg/ml) in 1% BSA/RPMI at 37°C for 1 hr and washed twice before layering 1 x 10^5^ cells onto 5μm transwell filters coated with 0.1 mg/ml fibronectin or a confluent layer of HS-5 cells. CXCL12 (160 ng/ml) or 1% BSA/RPMI media alone was added to the lower chamber. After 4 h incubation at 37°C and 5% CO2, cells migrating into lower chamber were enumerated using flow cytometry. Results were reported as the chemotaxis index: migration with chemokine divided by migration with media only.

### Xenogeneic NOD/SCID AML mouse model

NOD.CB17-*Prkdc*^*scid*^/J (NOD/SCID) female mice were purchased from the ARC. All mice were housed at the ANZAC Research Institute under specific pathogen free conditions. NOD/SCID mice were irradiated with 250cGy from an X-ray source (XRAD 320, Precision X-Ray; Connecticut, USA) one day before cell transfer. HL-60 cells were resuspended in X-VIVO at 2x10^6^ cells/ml and incubated with 10μg/ml MMRI-20 or isotype control mAb for 1 h. Antibody coated cells were washed three times with PBS and 5x10^6^ cells transferred intravenously (iv) into irradiated NOD/SCID mice. Mice were euthanised at pre-defined time point (d21) or at humane endpoint determined by disease score (maximum d28). To minimise suffering, disease scores were assessed daily for 14d post-transfer and then weekly if disease score ≤1, which derived from combined scores of 0–2 for weight loss, posture, activity and fur texture. A disease score of ≥4 led to euthanasia within an hour. No animal died prior to meeting defined endpoint. BM, spleen and blood were collected for flow cytometry analysis after euthanasia as described [12]. HL-60 cells were identified by flow cytometry using mouse CD45-PerCP/Cy5.5 (30-F11), human CD45-FITC (HI-30) and CD33-PE (WM53) mAbs with the gating strategy in panel B of S1 Fig. Engraftment was assessed as: number of human CD45 cells/ number of human plus mouse CD45 cells. All animal procedures and staff were approved by the Sydney Local Health District Animal Ethics Committee (#2015/026).

### Pyrrolobenzodiazepine (PBD) cytotoxicity assay

HL-60 and HepG2 cells (1x10^4^) or human PBMC (1x10^5^) were plated in quadruplicate with serial dilutions of MMRI-20 or isotype control and equimolar concentrations of GAM IgG secondary antibody attached via cleavable linker to PBD (Moradec). Cells were incubated at 37°C and 5% CO_2_ for 96h. Viability was then measured with CellTiter-Glo luminescent assay (Promega) for cell lines or flow cytometry for dendritic cells (DAPI^-^Lin^-^HLA-DR^+^CD11c^+^) and monocytes (DAPI^-^SSC^hi^Lineage^+^HLA-DR^+^CD11c^+^) in PBMC and compared to untreated samples.

### Statistics

Statistical analyses were performed with Prism 7 (GraphPad Software). Mean values with SEM are shown in graphs. Pearson’s coefficient (r) was used to determine correlation. Non-parametric paired or unpaired t-tests or ANOVA (with multiple test correction) were used to determine statistical differences between groups with *p*<0.05 deemed significant.

## Results

### CD302 is expressed on blasts and LSCs of most AML patients

CD302 has a restricted expression profile on myeloid cells within haematopoietic populations [11] leading us to hypothesise that this CLR could be expressed by AML that arise from the myeloid lineage. A comparison of *CD302* to *CD33* gene expression in a published cohort of 460 AML patients [13] demonstrated high expression of the former across FAB disease subtypes (highest on M4-M5) and a moderate positive correlation of expression between the markers (r = 0.4749, *p<*0.0001; panels A-B of S2 Fig).

**Fig 1:**
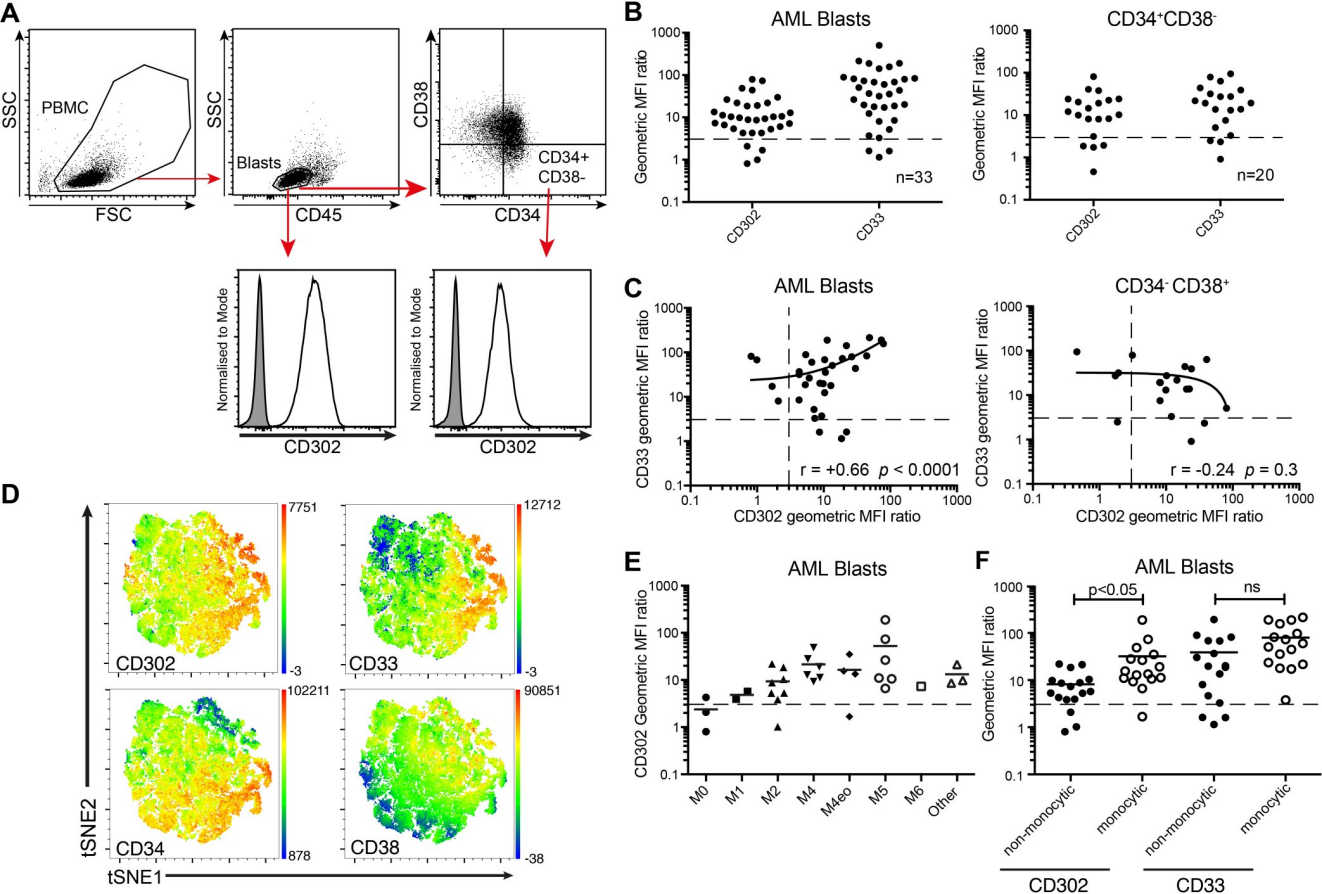
Expression of CD302 on leukemic blasts, CD34^+^CD38- LSC and HSC. (A) Gating strategy used to identify CD45^lo^SSC^lo^ AML blasts and CD34^+^CD38^-^ LSC. (B) Scatter dot plots showing the CD33 and CD302 expression on AML blasts (n = 33) and LSC fraction (n = 20). Samples were stained with MMRI-20 and CD33 mAb. Populations with a geoMFI ratio ≥3, shown above the dotted line, were considered to be positive. (C) Relationship between mean CD33 and CD302 expression on AML blasts and on AML LSC from patients. The solid lines were generated by linear regression. (D) Six parameter data (including CD117, CD34, CD33, CD38, CD45 and CD302) from five concatenated AML patient samples was converted into two t-SNE dimensions and overlayed with heatmaps of the indicated marker’s MFI. (E) Summary of CD302 expression by AML blasts across FAB subtypes based on their morphology and immunophenotypic characteristics, as outlined in the 2008 WHO classification. (F) CD302 and CD33 expression on AML samples with monocytic differentiation, FAB subtype M4 and M5, were compared to other subtypes of AML.

We examined a panel of primary AML blood samples of 33 patients with varied morphological and karyotypic classifications (S1 Table) for cell surface protein expression of CD302 by flow cytometry (Fig 1A) using MMRI-20. The geoMFI ratio of CD302 staining relative to a mouse IgG1 isotype control demonstrated that CD302 was expressed 3-fold higher than background on the surface of AML blasts in 88% of patients (29/33) and in LSC enriched CD34^+^CD38^-^ cells in 80% of patients (16/20; Fig 1B). This was similar to the proportion of AML cases showing surface staining of CD33 with the WM53 mAb clone (91% of blast and 85% of CD34^+^CD38^-^ cells). No significant difference was observed between the geometric MFI ratios of CD302 and CD33 in blasts or CD34^+^ CD38- AML in the patient cohort. Consistent with the gene expression analysis, correlation analysis revealed that mean CD302 expression correlated positively with mean CD33 expression on AML blasts in patient samples (r = 0.66, *p*<0.0001; Fig 1C). However, no significant correlation between CD302 and CD33 was observed in the CD34^+^CD38^-^ populations. T-SNE is an algorithm that reduces multi-parameter flow cytometry data into two t-SNE parameters, allowing visualization of cellular organisation in two dimensions. Transforming the six parameter data (including CD33, CD34, CD38, CD45, CD117 and CD302 expression) from five concatenated AML samples into a two dimensional t-SNE plot and highlighting areas of high CD302 and CD33 expression illustrated the correlation between these markers at the cellular level (Fig 1D). As CD302 is highly expressed by human blood monocytes [12], we examined if AML with monocytic differentiation would express a higher amount of CD302. Indeed, CD302 was expressed significantly higher in AML from these patients than those with non-monocytic subtype cases (Fig 1E and 1F).

### MMRI-20 is internalised and mediates ADCC against leukemic cell lines

**Fig 2:**
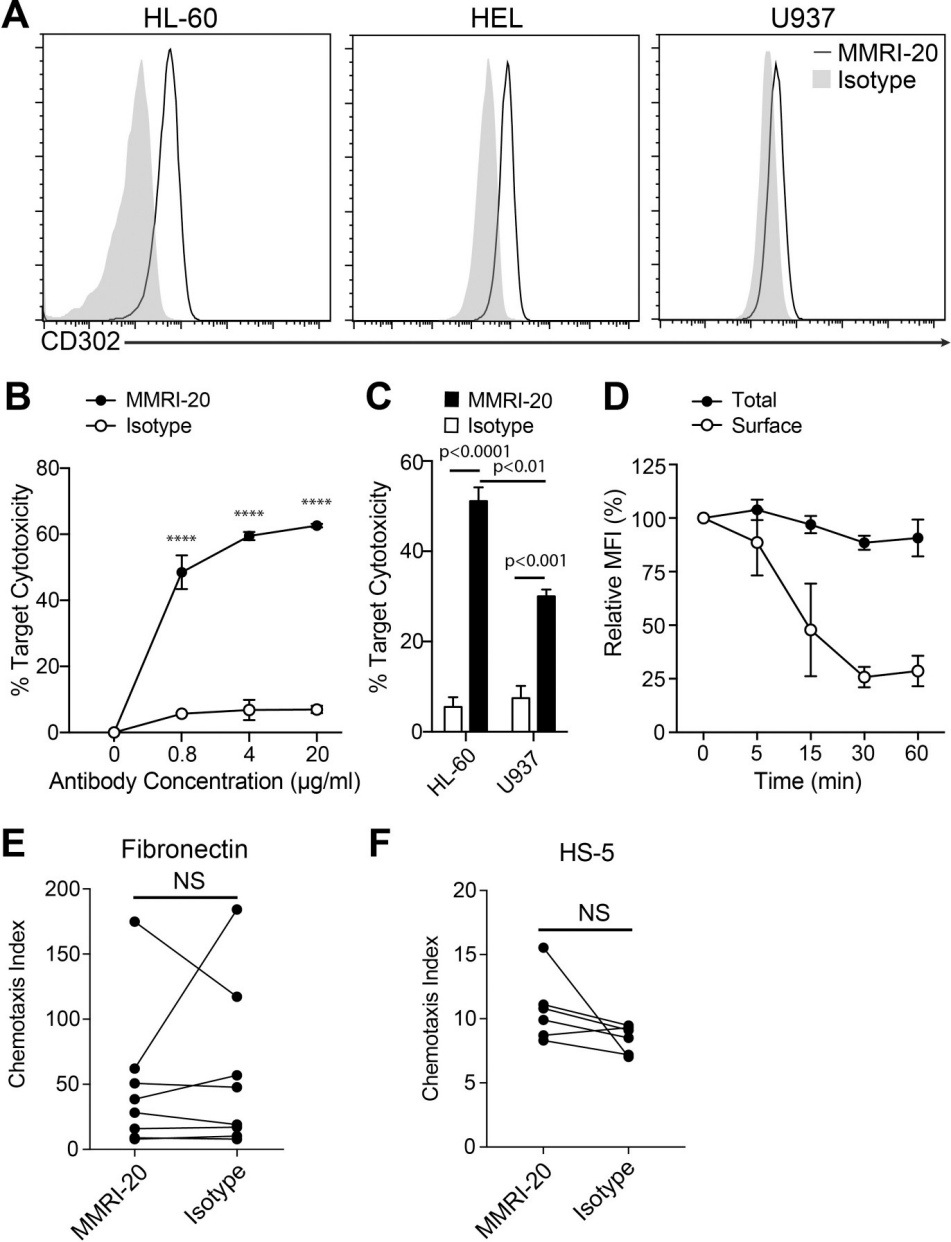
Antibodies targeting CD302 are able to be internalised and mediate ADCC of target cells. (A) Flow cytometry histograms showing the surface expression of CD302 on leukemic target cell lines as determined by staining with the MMRI-20 compared to a mouse IgG1 isotype control. (B) CD302 internalisation by HL-60 cells determined by flow cytometry. Total staining determined by MMRI-20-FITC 37°C incubation for the indicated times after which residual surface CD302 was measured with anti-mouse IgG-PE at 4°C. MFI of antibody staining is reported relative to pre-incubation levels. (C) MMRI-20 induced ADCC against HL-60 target cells. Calcein-AM labelled HL-60 were incubated for 18h with mouse spleen effectors at a 1:10 ratio, together with1000U IL-2 and the indicated concentrations of MMRI-20 or isotype control mAb. Target cell killing was measured as 7-AAD^+^ Calcein-AM^+^ cells by flow cytometry and presented relative to death in target alone (0%) or with 2% Triton X solution (100%). **** p<0.0001, two-way ANOVA. (D) ADCC elicited against HL-60 (CD302^hi^) and U937 (CD302^lo^) leukemic targets using 20μg/ml MMRI-20 or isotype mAb control. Experiments representative of three experiments. Differences tested by two-way ANOVA. (E-F) HL-60 cells were incubated with either MMRI-20 or isotype control mAb for 30 mins at 37°C and tested for their ability to migrate across 5 μm transwells coated with (E) fibronectin or (F) HS-5 cells towards 160 ng/ml CXCL12 or media alone. Cells in bottom chamber were enumerated after 4h by using flow cytometry and migration presented as the chemotaxis index. Circles connected by lines represent individual paired experiments. No significant difference (NS) between MMRI-20 and isotype group (paired t-test).

We next investigated whether targeting CD302 with a mAb could be therapeutic against AML. Three potential target leukemic cells lines, HL-60, HEL and U937 were examined for their surface CD302 expression by flow cytometry. MMRI-20 staining was highest on HL-60, followed by HEL and lowest on U937 (geoMFI ratios of 5.4, 3.3 and 1.6, Fig 2A), consistent with previously described transcript levels [15].

To determine whether targeting surface CD302 with mAb could mediate ADCC towards leukemic cells by immune effector cells, HL-60 were co-cultured with mouse splenocytes as effector cells in the presence of different concentrations of MMRI-20 or an isotype control mAb. After 20 h of culture, addition of MMRI-20 increased the killing of target cells in a dose dependent manner, while culturing with the isotype control resulted in minimal killing (Fig 2B). We compared ADCC mediated against surface CD302^hi^ HL-60 to that of CD302^lo^ U937 with optimal levels of antibody. As anticipated, target killing was greater for HL-60 than U937 (Fig 2C). However, despite low surface CD302 levels, ADCC against U937 induced by MMRI-20 was still significantly higher than that achieved by the isotype control.

The internalising capacity of MMRI-20 was tested on the CD302^hi^ HL-60 cell line using a flow cytometry based assay (Fig 2D). The assay showed that MMRI-20 bound to the surface of cells was reduced to ~75% of its starting level after 30 min of culture at 37°C (Fig 2D). In contrast, the total level of MMRI-20 (surface and intracellular) remained constant through the course of the experiment, indicating that the antibody had not dissociated from the surface but was internalised into cells with CD302 (Fig 2D).

Given that we have previously shown CD302 to contribute to migration [12], we explored whether MMRI-20 could alter this function when bound to AML cells. We performed an *in vitro* assay where the chemotactic ability of HL-60 to the BM homing chemokine CXCL12 was compared between cells pre-incubated with MMRI-20 or an isotype control antibody (Fig 2D). Transwells were coated with either fibronectin or HS-5 stromal cells, forcing cells to utilise cellular protrusion (e.g. podosomes) to transmigrate through the barrier. Regardless, HL-60 migrated in a similar fashion towards CXCL12 in all conditions tested, suggesting MMRI-20 did not alter the chemotactic ability of the leukemic cells, at least in this *in vitro* assay.

### Anti-CD302 mAb reduces engraftment of AML in NOD/SCID mice but does not alter survival

**Fig 3:**
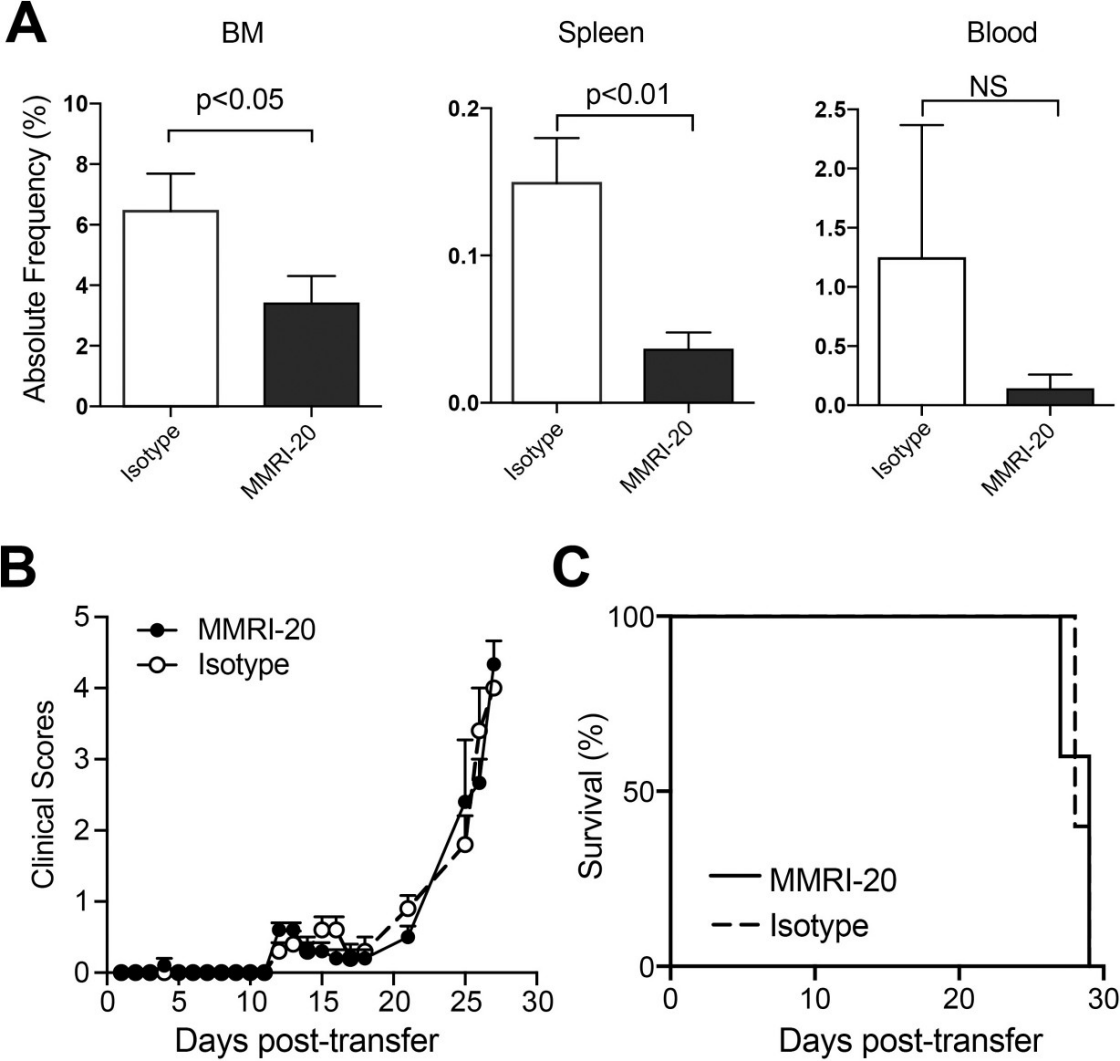
*Ex vivo* MMRI-20 binding reduces engraftment of leukemic cell lines HL-60 in NOD/SCID mice. (A) Bar graphs showing absolute frequency of HL-60 cells coated with MMRI-20 or isotype control mAb in BM, spleen and blood of NOD/SCID mice 21 days after iv injection (n = 6/group). HL-60 cells were identified as human CD33^+^, human CD45^+^, mouse CD45^-^ cells in tissue cell suspensions by flow cytometry (see panel B of S1 Fig). (B) Disease scores and (C) survival curves of five NOD/SCID mice injected with MMRI-20 or isotype control mAb coated HL-60 cells.

We established a xenogeneic model of AML for testing antibody targeting. HL-60 cells were injected iv into irradiated NOD/SCID mice, allowing leukemic cells to engraft and disseminate causing illness requiring euthanasia between d21 to 28 post cell transfer. To investigate whether anti-CD302 antibodies affected AML engraftment, we pre-coated HL-60 cells with MMRI-20 or an isotype control *ex vivo* prior to transferring them into NOD/SCID mice. Both cohorts were euthanised on d21 and the presence of AML in their BM, spleen and blood was examined by flow cytometry. Coating with MMRI-20 significantly reduced HL-60 burden in BM and spleens and a trend towards reduction in the blood (Fig 3A). However, this difference did not lead to a lower disease score nor an extended survival time for mice receiving MMRI-20 versus isotype control coated AML (Fig 3B and 3C).

### CD302 is expressed by healthy HSC in BM and CB

**Fig 4:**
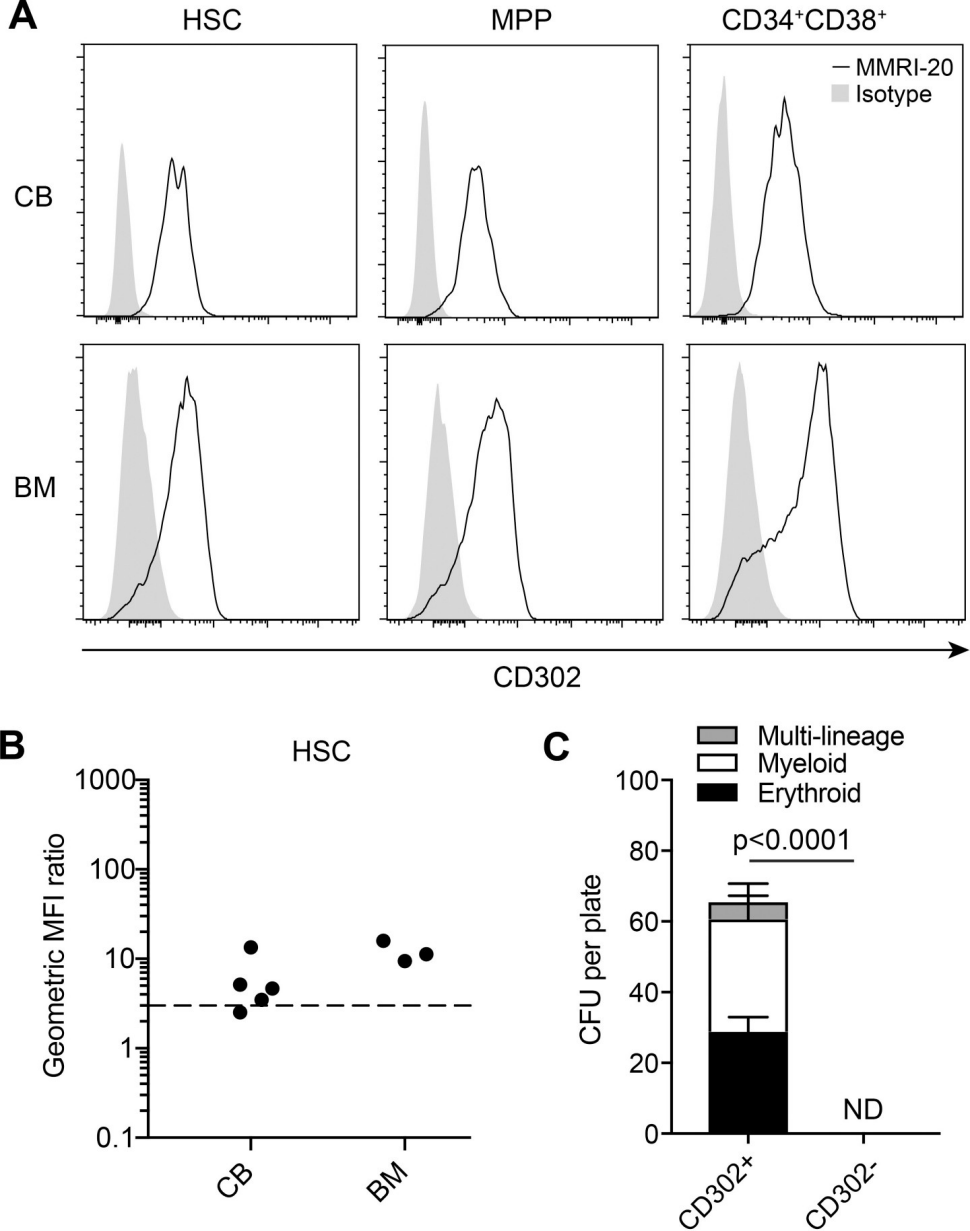
CD302 is expressed on healthy BM and cord blood. (A) Representative flow cytometry histograms showing the expression of CD302 on healthy BM (n = 3) and CB (n = 5) HSC, MPP and CD34^+^CD38^-^ populations. Gating strategy shown in panel A of S1 Fig. (B) geoMFI ratios of CD302 expression on HSC in all BM and CB samples. Dotted line indicates a geoMFI of 3 above which was considered positive. (C) Graphs showing CFU of multi-lineage, myeloid and erythroid lineages counted 12–14 days after seeding wells with equal numbers of Lin^-^CD302^+^ and CD302^-^ sorted CB populations (n = 6). ND, no detection of CFU colonies.

We obtained three BM and five cord blood samples from healthy donors and examined CD302 expression on HSC and progenitors via flow cytometry (Fig 4A). MMRI-20 was found to bind to the vast majority of HSC, MPP and CD34^+^CD38^+^ from both BM and cord blood in all samples (Fig 4A and 4B). To determine whether CD302^-^ progenitor populations could maintain normal haematopoiesis, we compared the CFU potential of CD302^+^ or CD302^-^ populations. The two progenitor fractions were isolated by FACS based on MMRI-20 staining and CFU of various blood developmental lineages deriving from each set were compared. Generation of CFU of all lineages were completely abrogated in the CD302^-^ fraction (Fig 4C and 4D), suggesting that the majority of HSC progenitors were contained in the CD302^+^ fraction.

### Characterisation of CD302 expression by liver cells

Expression of CD302 in liver was previously reported [11, 12]. We compared the transcriptional expression of *CD302* in human liver, CD14^+^ monocytes, HepG2, HL-60, and U937 using quantitative PCR (panel A of S3 Fig). Consistent with our previously studies [11, 12], liver showed the highest expression of *CD302* amongst the tested samples. We then investigated if the MW of human CD302 protein in liver was different to that of leukemic cells by Western blot (panel B of S3 Fig). In contrast to the mouse studies, CD302 in the human liver cell line HepG2 showed a similar MW to that in the HL-60 leukemic cell line. When HepG2 was labelled with MMRI-20 and examined by flow cytometry or immunohistology, we observed that that CD302 protein was primarily intracellular with minimal expression on the cell surface (panel C-D of S3 Fig). This was in contrast to the MMRI-20 staining pattern of the leukemic HL-60 cell line, where surface CD302 staining was detected by both techniques.

### PBD toxin delivery through CD302 mAb mediates killing of leukemic but not hepatic cell lines

**Fig 5:**
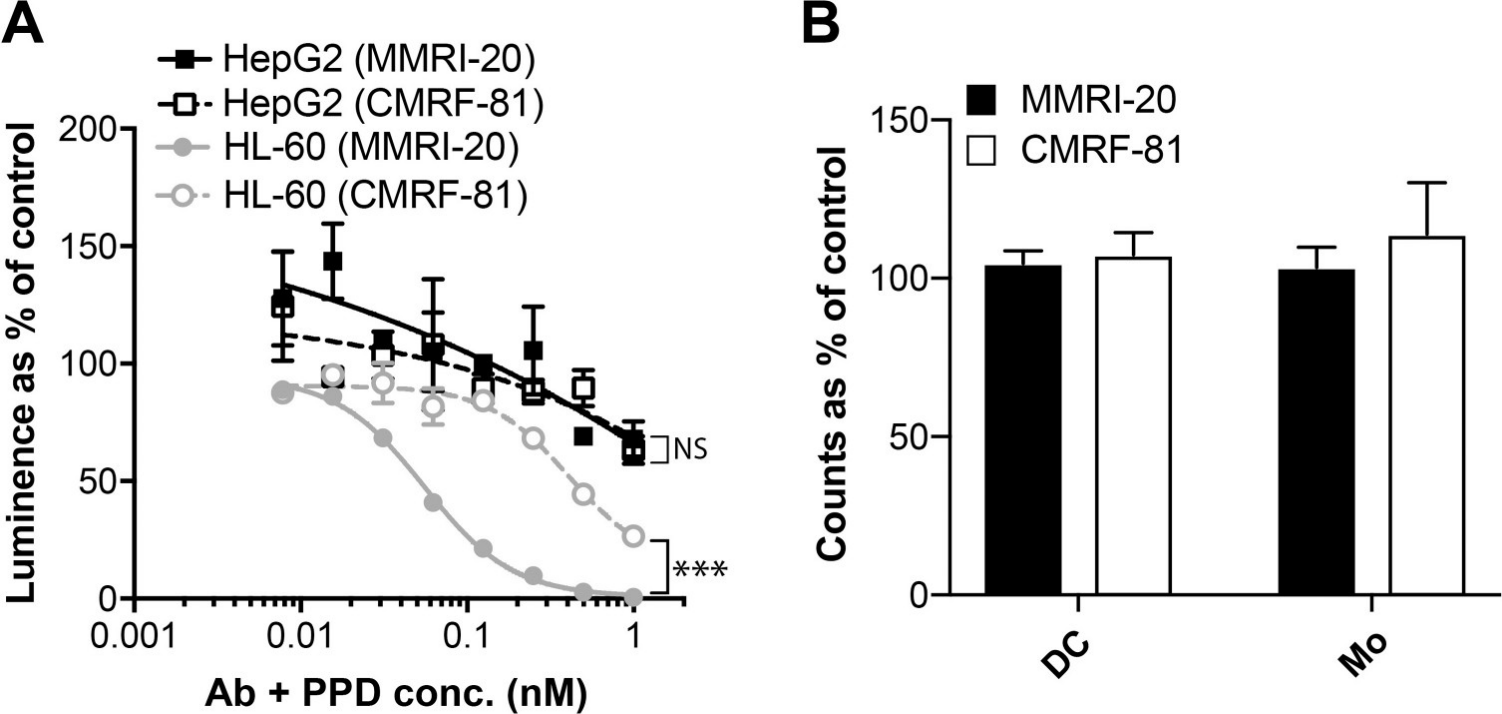
PBD delivery through CD302 mAb mediates killing of leukemic but not hepatic cell lines. (A) Comparison of HepG2 or HL-60 killing after 96 h culture with the indicated concentrations of MMRI-20 or isotype control mAb together with equimolar concentrations of a GAM IgG secondary antibody conjugated with PBD toxin. Cell viability measured with the CellTiter-Glo luminescent assay and compared as a % to untreated controls. One of three representative experiments shown. ***p<0.001, 2-way ANOVA. (B) PBMC were cultured in quadruplicate for 96h with 1nM MMRI-20 or isotype control mAb together with equimolar concentrations of a GAM IgG secondary antibody conjugated with PBD toxin. Flow cytometry was used to count viable (DAPI^-^) dendritic cells (Lin^-^ HLA-DR^+^CD11c^+^) and monocyte (SSC^hi^Lineage^+^HLA-DR^+^CD11c^+^) in wells after culture and compared as a % to untreated controls. One of two representative experiments shown.

To examine the potential of CD302 as an ADC target, HL-60 or HepG2 cells were co-cultured with either MMRI-20 or isotype control antibody in the presence of GAM IgG mAb bound to PBD (GAM-PBD). PBD delivered high toxicity towards HL-60 via MMRI-20 in a dose dependent manner when compared to the isotype control (Fig 5A). In contrast, HepG2 co-cultured with either MMRI-20 or isotype antibody plus GAM-PBD both showed equivalent, minimal toxicity, suggesting that the killing of HepG2, was not CD302 specific (Fig 5A). Similarly, we found no toxicity towards dendritic cells or monocytes when PBMC were cultured with MMRI-20 or isotype antibody plus GAM-PBD for 96h (Fig 5B).

## Discussion

Immunotherapies involving mAbs and their derivatives have proven to be encouraging strategies for treating haematological malignancies. There is a pressing need for alternative targets for AML treatment, especially those expressed by LSC that cause disease relapse. We have identified the CLR CD302 as a possible AML target. Using the mouse anti-human CD302 mAb MMRI-20, we showed expression of CD302 on primary AML blast and various leukemic cell lines. The vast majority of primary AML samples expressed CD302 (88%) with significant correlation to CD33 (a clinically established target for AML) at the transcript, cellular and patient level. Importantly, MMRI-20 bound to CD34^+^CD38^-^ blasts, a population enriched in LSC, in 80% of patient samples.

There are various therapeutic mechanisms of an anti-AML mAb including ADCC through recruitment of immune effector cells or through inhibition of critical functions of AML [5]. MMRI-20 could mediate the ADCC of leukemic cell lines expressing high or low levels of CD302. In spite of the role for CD302 in migration [11, 12], binding of MMRI-20 to HL-60 leukemic cells did not alter their mobilisation towards the BM-homing chemokine CXCL12 in fibronectin or stromal cell transmigration assays. However, we cannot rule out that the antibody could inhibit migration in other experimental settings. Future identification of the ligand for CD302 will provide insight into whether antibodies can block ligand interaction and how this alters AML migration.

Further investigation into the therapeutic capability of the MMRI-20 mAb for AML was performed in an *in vivo* xenogeneic NOD/SCID model. Binding of CD302 with MMRI-20 reduced engraftment of the HL-60 leukemic cell line in BM, spleen and blood. However, the ADCC function of the naked mouse antibody in this model was likely hampered by the low natural killer and macrophage activity in NOD/SCID mice [16], which may explain why HL-60 was not completely eliminated from hosts and why this did not lead to a survival advantage.

A common strategy for improving the therapeutic activity of anti-cancer antibodies is to attach a toxin or radioisotope for delivery to target cells (i.e. ADC). Like other CLR, CD302 is endocytic and was previously shown to take up MMRI-20 antibodies in CD302 transfected Chinese hamster ovary cells and healthy blood myeloid cells [11]. The rapid internalisation of MMRI-20 by the HL-60 leukemic cell line denoted CD302’s potential as a target for ADC. Evidence for the likely efficacy of a CD302 ADC was provided with the efficient killing (sub-nanomolar IC50) of HL-60 cells using a secondary mAb to deliver PBD via MMRI-20 internalisation.

Target molecules for AML to date have unavoidably shown wider expression on healthy myeloid populations [5]. This is also the case with CD302, which can be found on monocytes, macrophages, granulocytes and dendritic cells [11, 12]. MMRI-20 was also shown to bind HSC in healthy BM and cord blood samples. Although the presence of CD302 on healthy haematopoietic cells might cause potential toxicity against these populations, studies have shown that ADC targeting markers (e.g. gemtuzumab ozogamicin with CD33) expressed by HSC progenitors and myeloid populations [17] can still provide clinical benefits for patients [18]. The presence of CD302 on HSC would therefore not necessarily exclude it as a potential therapeutic target. Haematological toxicity could be managed by adding CD302 mAb or ADC treatment to conditioning therapies given prior to HSCT.

Another consideration for utilising CD302 as an AML target is its expression in liver, raising concerns of hepatotoxicity [11]. Mouse studies have shown that CD302 transcript is expressed by hepatocytes and liver sinusoidal endothelial cells [12], although the liver protein appeared to exhibit a different MW to that in myeloid cells. Western blot analysis of the human liver cell line HepG2 and leukemic cell line HL-60 showed a similar MW for CD302. However, in contrast to the abundant surface CD302 found on HL-60, we predominantly detected intracellular distribution on the HepG2 cell line with undetectable surface expression by flow cytometry or immunohistology. Consistent with this difference, delivery of PBD via CD302 could kill HL-60 but not HepG2, raising the possibility that a CD302 ADC could deliver a therapeutic effect against AML cells with minimal liver toxicity. It is noted that this observation needs confirmation in primary liver cells and further study into CD302 function in this organ is required.

## Conclusion

The heterogeneous nature of AML and the urgent need for new therapeutics makes characterising all potential target markers a necessity. CD302 is expressed highly on blasts and LSC enriched CD34^+^ CD38^-^ populations in the majority of AML patients thereby showing potential as a therapeutic AML target. Further studies are required to investigate the potential toxicity of a CD302 mAb and derivatives in healthy human tissue and establish the ideal therapeutic window for their use.

## Supporting information

S1 Table Clinical and pathological characteristics of AML patient samples tested in the current study. (DOCX)

S1 Fig Gating strategies. (A) Gating used to identify progenitor cells in human BM and CB samples (CB shown). Gating strategies to identify HL-60 leukemic cells in NOD/SCID BM, spleen and blood (BM shown).
(TIF)

S2 Fig Microarray analysis of transcript levels of CD302 and CD33 in AML patients. (A) Probes specific for *CD302* and *CD33* transcripts were compared in a cohort of 460 AML patients over various FAB subtypes. Statistics shown in table below. (B) Correlation of CD302 and CD33 gene expression in all patients.
(TIF)

S3 Fig CD302 is expressed minimally on the surface of human liver cells. (A) Transcript expression of CD302 relative to the HPRT housekeeping gene was determined by qPCR in three cDNA samples derived from human liver, monocytes or the indicated cell lines. Expression shown as fold changes relative to the U937. (B) Western blot comparing the size of CD302 protein band in HepG2 and HL-60 cells. (C) Comparison of flow cytometry CD302 surface staining of HepG2 and HL-60 cell lines with MMRI-20 compared to an isotype control. (D) Immunohistology staining of CD302 (green) with MMRI-20 in HepG2 or HL-60 cells. Phalloidin staining (red) was used to highlight the cellular surface while DAPI (blue) staining reveals the nucleus. A composite of phalloidin and DAPI with MMRI-20 or isotype control antibody staining is shown for comparison. Scale bar marks 20μm.
(TIF)
