# Supplementary material for: Examination of CD302 as a potential therapeutic target for acute myeloid leukemia
Source: PLoS One. 2019 May 10;14(5):e0216368. doi: 10.1371/journal.pone.0216368 (PMC6510432; doi:10.1371/journal.pone.0216368)
Supplement: S1 Table — (DOCX) [file pone.0216368.s001.docx]

**Supplementary Table 1.** Clinical and Pathological Characteristics of AML Patient Samples Tested in the Current Study.

| Age | Sex | Sample | WHO Diagnosis | FAB | WCC | BM blasts % | Karyotype | NPM1 | FLT3-ITD |
| --- | --- | --- | --- | --- | --- | --- | --- | --- | --- |
|  |  |  |  |  |  |  |  |  |  |
| 23 | M | BM | AML with inversion 16 | M4Eo | 160 | 60 | 46,XY,inv(16)(p13;q22) | Negative | Negative |
| 44 | F | PB | AML with inversion 16 | M4Eo | 35 | 50 | Inversion 16 | Negative | Negative |
| 51 | UD | BM | AML with inversion 16 | M4Eo | UD | UD | Inversion 16 | NT | NT |
| 16 | M | BM | AML with inversion 16 | M4Eo | 111 | 45 | Inversion 16 | NT | NT |
| 76 | F | BM | AML not otherwise specified | M4 | 14 | 70 | Normal | NT | NT |
| 74 | M | PB | AML not otherwise specified | M2 | 16 | 43 | +8 | NT | NT |
| 59 | M | PB | AML not otherwise specified | M5 | 146 | 95 | Normal | NT | NT |
| 51 | M | PB | AML not otherwise specified | M0 | 2.5 | 73 | No metaphases | Negative | Negative |
| 86 | F | PB | AML not otherwise specified | M1 | 23 | NT | unknown | NT | NT |
| 61 | F | BM | AML not otherwise specified | M6 | 0.7 | 26 | complex | NT | NT |
| 41 | F | BM | AML not otherwise specified | M5 | 5.2 | 78 | complex | NT | Negative |
| 59 | M | BM | AML not otherwise specified | M0 | 14 | 60 | Normal | Negative | Negative |
| 24 | M | PB | AML not otherwise specified | M5 | 64 | 90 | t(11;17), +8 | Negative | Negative |
| 73 | F | PB | AML not otherwise specified | M0 | 8 | 85 | Normal | Detected | Negative |
| 79 | M | BM | AML not otherwise specified | M2 | 55 | 60 | +6 | Negative | Negative |
| 61 | M | BM | AML not otherwise specified | M2 | 7 | 25 | No metaphases | NT | NT |
| 44 | UD | BM | AML not otherwise specified | M1 | UD | UD | t(9:11) | NT | NT |
| 86 | UD | BM | AML not otherwise specified | M4 | UD | UD | +8 | NT | NT |
| 19 | UD | BM | AML not otherwise specified | M2 | UD | UD | monosomy 7 | NT | NT |
| 61 | UD | BM | AML not otherwise specified | M4 | UD | UD | Normal | NT | NT |
| 77 | F | PB | AML with myelodysplasia-related changes | M2 | 128 | 55 | Normal | NT | NT |
| 89 | M | PB | AML with myelodysplasia-related changes | M2 | 30 | 16 | Normal | NT | NT |
| 59 | M | PB | AML with myelodysplasia-related changes | M4 | 54 | 56 | Normal | Negative | Detected |
| 95 | F | PB | AML with myelodysplasia-related changes | M5 | 63 | NT | unknown | NT | NT |
| 82 | M | PB | AML with myelodysplasia-related changes | M5 | 3.8 | 53 | +19 | NT | NT |
| 75 | M | PB | AML with myelodysplasia-related changes | M2 | 6.5 | 60 | Normal | NT | NT |
| 72 | F | PB | AML with myelodysplasia-related changes | M5 | 30 | 80 | Complex;del(12)(p12),del(13)(q21), add(17)(p11.2)[1]46,XX[1] | NT | NT |
| 70 | M | BM | Therapy related myeloid neoplasm | M4 | 16 | 82 | Normal | Detected | Detected |
| 79 | M | PB | Therapy related myeloid neoplasm | M4 | 150 | 67 | 46, XY, add(12)(q24.3) | NT | NT |
| 87 | M | PB | Therapy related myeloid neoplasm | M2 | 2.2 | 62 | 47,XY,t(8;16)(p11;p13), +8 | NT | NT |
| 75 | M | PB | Myelodysplastic syndrome with excess blasts | N/A | 3.5 | 15 | +8 | NT | NT |
| 81 | M | PB | Chronic myelomonocytic leukemia | N/A | 5.7 | 10 | Normal | NT | NT |
| 55 | M | BM+PB | Blastic plasmacytoid dendritic cell neoplasm | N/A | 4.3 | 85 | Normal | Negative | Negative |
