# Supplementary figures and images for: Examination of CD302 as a potential therapeutic target for acute myeloid leukemia
Source: PLoS One. 2019 May 10;14(5):e0216368. doi: 10.1371/journal.pone.0216368 (PMC6510432; doi:10.1371/journal.pone.0216368)

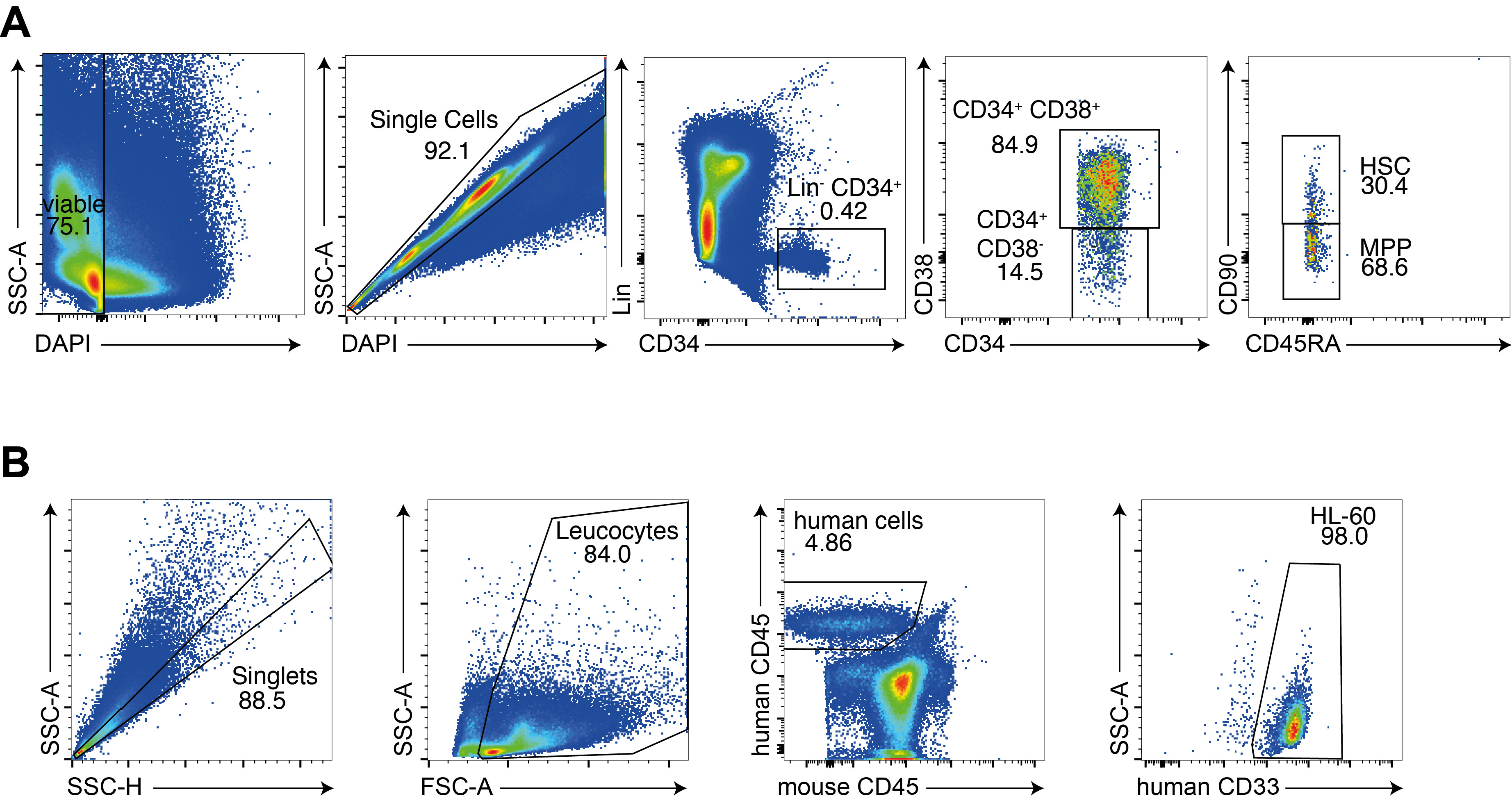

Supplement: S1 Fig — (A) Gating used to identify progenitor cells in human BM and CB samples (CB shown). Gating strategies to identify HL-60 leukemic cells in NOD/SCID BM, spleen and blood (BM shown). (TIF) [file pone.0216368.s002.tif]

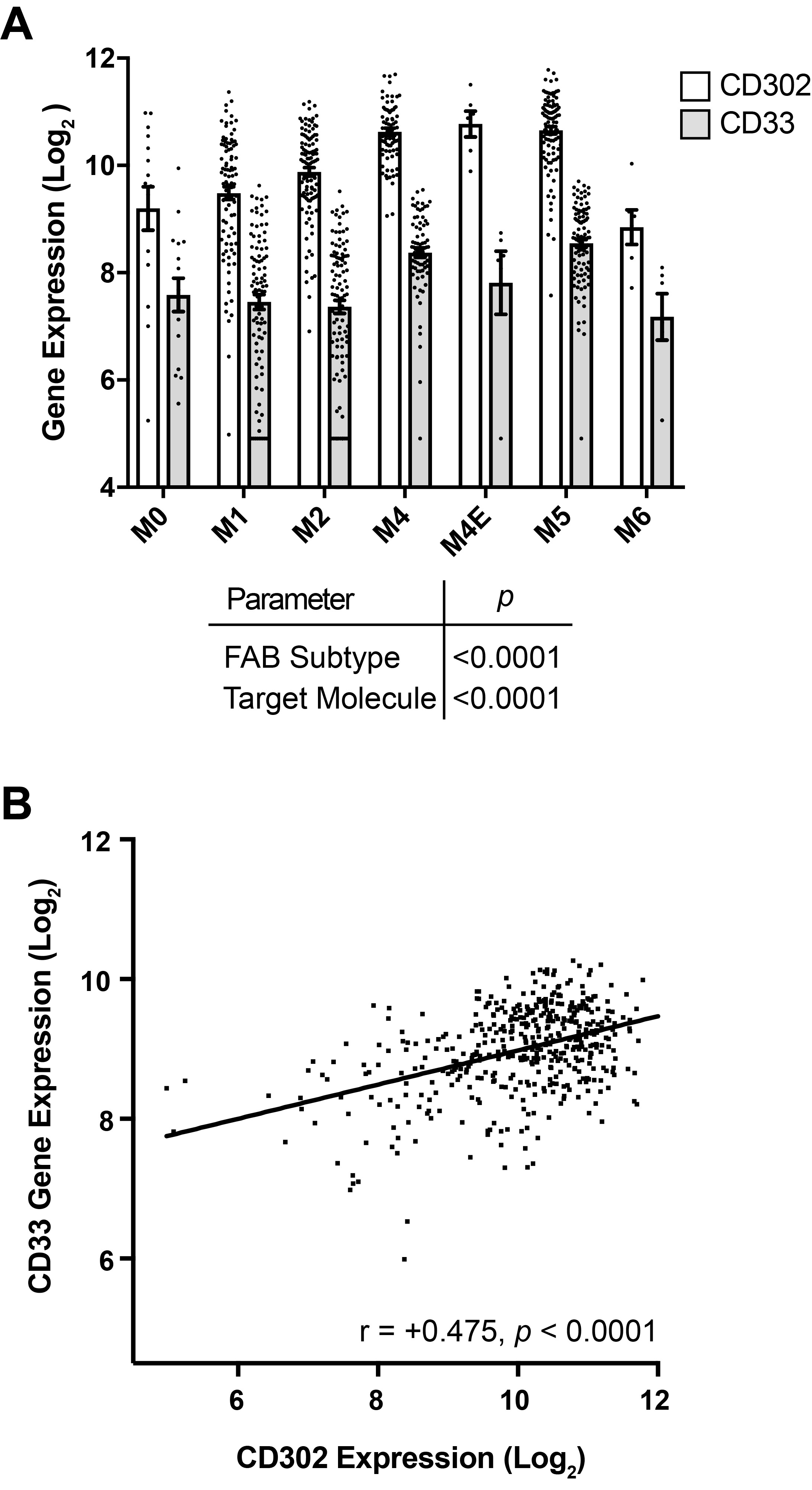

Supplement: S2 Fig — (A) Probes specific for CD302 and CD33 transcripts were compared in a cohort of 460 AML patients over various FAB subtypes. Statistics shown in table below. (B) Correlation of CD302 and CD33 gene expression in all patients. (TIF) [file pone.0216368.s003.tif]

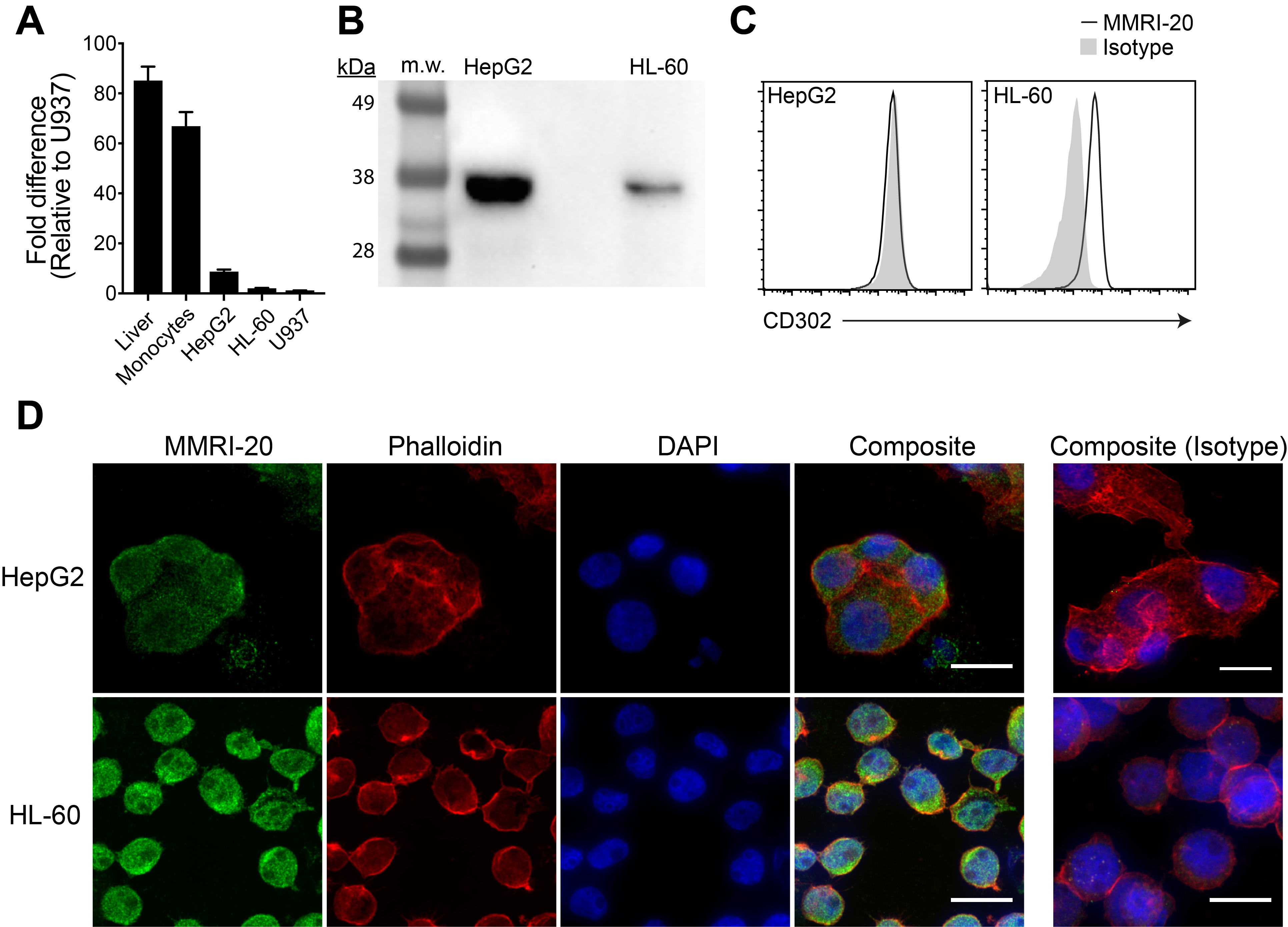

Supplement: S3 Fig — (A) Transcript expression of CD302 relative to the HPRT housekeeping gene was determined by qPCR in three cDNA samples derived from human liver, monocytes or the indicated cell lines. Expression shown as fold changes relative to the U937. (B) Western blot comparing the size of CD302 protein band in HepG2 and HL-60 cells. (C) Comparison of flow cytometry CD302 surface staining of HepG2 and HL-60 cell lines with MMRI-20 compared to an isotype control. (D) Immunohistology staining of CD302 (green) with MMRI-20 in HepG2 or HL-60 cells. Phalloidin staining (red) was used to highlight the cellular surface while DAPI (blue) staining reveals the nucleus. A composite of phalloidin and DAPI with MMRI-20 or isotype control antibody staining is shown for comparison. Scale bar marks 20μm. (TIF) [file pone.0216368.s004.tif]
